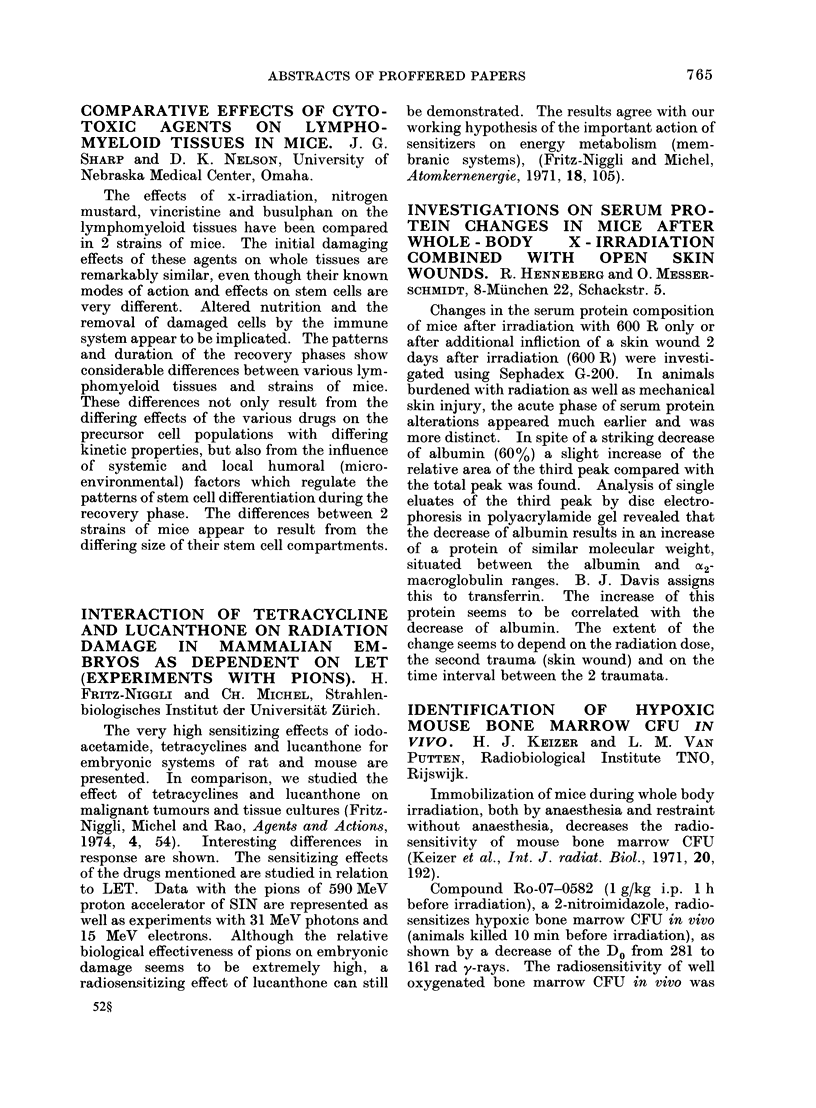# Proceedings: Comparative effects of cytotoxic agents on lymphomyeloid tissues in mice.

**DOI:** 10.1038/bjc.1975.334

**Published:** 1975-12

**Authors:** J. G. Sharp, D. K. Nelson


### COMPARATIVE EFFECTS OF CYTOTOXIC AGENTS ON LYMPHOMYELOID TISSUES IN MICE.

J. G. SHARP and D. K. NELSON, University of Nebraska Medical Center, Omaha.

The effects of x-irradiation, nitrogen mustard, vincristine and busulphan on the lymphomyeloid tissues have been compared in 2 strains of mice. The initial damaging effects of these agents on whole tissues are remarkably similar, even though their known modes of action and effects on stem cells are very different. Altered nutrition and the removal of damaged cells by the immune system appear to be implicated. The patterns and duration of the recovery phases show considerable differences between various lymphomyeloid tissues and strains of mice. These differences not only result from the differing effects of the various drugs on the precursor cell populations with differing kinetic properties, but also from the influence of systemic and local humoral (microenvironmental) factors which regulate the patterns of stem cell differentiation during the recovery phase. The differences between 2 strains of mice appear to result from the differing size of their stem cell compartments.